# Relationship Study on Land Use Spatial Distribution Structure and Energy-Related Carbon Emission Intensity in Different Land Use Types of Guangdong, China, 1996–2008

**DOI:** 10.1155/2013/309680

**Published:** 2013-02-07

**Authors:** Yi Huang, Bin Xia, Lei Yang

**Affiliations:** ^1^Sustainable Development Research Center, Guangzhou Institute of Geochemistry, Chinese Academy of Sciences, Guangzhou 510640, China; ^2^University of Chinese Academy of Sciences, Beijing 100049, China; ^3^School of Marine Sciences, Sun Yat-Sen University, Guangzhou 510006, China; ^4^College of Resources and Environment, Guangdong University of Business Studies, Guangzhou 510320, China

## Abstract

This study attempts to discuss the relationship between land use spatial distribution structure and energy-related carbon emission intensity in Guangdong during 1996–2008. We quantized the spatial distribution structure of five land use types including agricultural land, industrial land, residential and commercial land, traffic land, and other land through applying spatial Lorenz curve and Gini coefficient. Then the corresponding energy-related carbon emissions in each type of land were calculated in the study period. Through building the reasonable regression models, we found that the concentration degree of industrial land is negatively correlated with carbon emission intensity in the long term, whereas the concentration degree is positively correlated with carbon emission intensity in agricultural land, residential and commercial land, traffic land, and other land. The results also indicate that land use spatial distribution structure affects carbon emission intensity more intensively than energy efficiency and production efficiency do. These conclusions provide valuable reference to develop comprehensive policies for energy conservation and carbon emission reduction in a new perspective.

## 1. Introduction

Since energy-related carbon emission was recognized as the most important anthropogenic factor that leads to global warming [1, 2], numerous studies have focused on the mechanism of how human activities affect energy consumption. Economic development, as well as industrial structure and energy consumption structure are typically considered as primary influencing factors for energy consumption [3–7]. Actually, human activity affects the regional carbon emission through changing the land use patterns, which in turn changes the energy consumption patterns and finally influences the amount and rate of carbon emission [8–10]. However, studies on land use change-induced carbon emission are usually directed toward calculating carbon storage in soil and vegetation cover given that soil respiration also considerably accounts for carbon dioxide emission into the atmosphere [11–20]. The social attribution of land use, which affects energy consumption via the spatial distribution of land use, is always neglected.

Related research focuses on the relationship between urban form and energy consumption [21, 22]. The majority of such research suggests a strong negative correlation between urban density and energy consumption [23–28]. Concentration distribution reduces energy consumption, resulting in the emission of fewer pollutants and maintenance of sustainable urban development, particularly for traffic and residential lands [29–37]. This view is accepted by policy makers in many developed countries, and is considered as an effective way to reduce energy consumption [26, 27]. Opposing perspectives, however, also exist. For example, Mindali et al. (2004) argues that the relationship between urban form and energy consumption is uncertain [26]. Holden and Norland (2005) indicated that decentralized concentration may reduce energy consumption in households [38]. Ma and Jin (2011) found a weak correlation between urban density and energy consumption under high population density in China [39].

Urban form, defined on the basis of the spatial distribution of land use, encompasses urban size, population density, and other related elements [40]. Except for traffic land and residential land distribution, other types of land use are seldom regarded as influencing factors for energy consumption in existing research. Thus, the existed urban form perspective is an unsuitable approach to determining the relationship between the distribution of different land use types and energy-related carbon emission. A comprehensive relation study between the spatial distribution structure of different land use types and energy-related carbon emission should be considered at a regional scale, specific to domestic conditions in terms of the large population, defective infrastructure, and binary structures of urban and rural areas. 

Some researchers analyzed the energy-related carbon emission caused by land use patterns change in China, but none of them studied on their relationships. Zhao and Huang (2010) found that the energy-related carbon emission per unit land area in Jiangsu Province increased from 8.24 t/hm^2^ to 15.53 t/hm^2^ from 2003 to 2007, and that the highest carbon emission intensities originated from residential and industrial land [8]. Zhao et al. (2010) studied the energy-related carbon emissions of different industrial spaces in China [41]. The results indicate that living and industrial-commercial spaces contribute the highest carbon emissions, and that the carbon emission intensities of each industrial space in Shanghai are the highest among the investigated 30 provinces. Using data envelopment analysis, You and Wu (2010) measured the provincial energy-related carbon emission efficiency of land use [42]. The results show that carbon emission per unit land area is effective in only three provinces. Zhang (2011) analyzed the land use constitution structure in Yunnan Province through information entropy method, and confirmed that the changes in land use structure cause energy-related carbon emission in the province [43].

**Figure 1:**
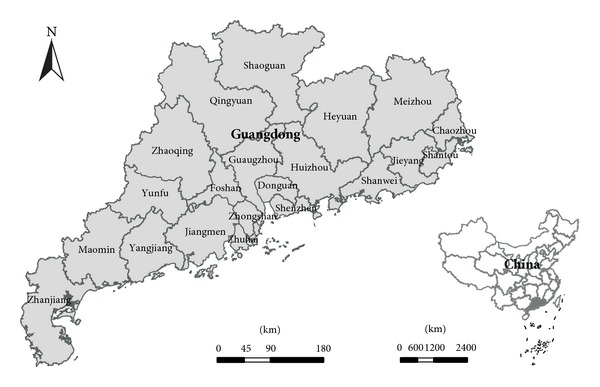
Geographic location and component cities of Guangdong Province in China.

Guangdong, which located in south China and comprises 21 cities (Figure 1), is one of the most developed provinces in the country. Its rapid development is attributed to the reform and opening policy implemented in 1978. Statistical data (taken from the Guangdong Statistical Yearbook and the Guangdong Agricultural Statistical Yearbook) show that Guangdong's GDP increased from 18.58 billion Yuan to 4601.31 billion Yuan from 1978 to 2010, and that its contribution to China's GDP increased from 5.1% to 11.47% during the same period. The proportions of output value in three major industrial sectors changed from 29.8% : 46.6% : 23.6% to 5% : 50% : 45%. The permanent population increased from 50.64 million to 104.40 million. In addition, land use spatial distribution and energy consumption significantly changed as human activities increased. Crop land decreased from 6.6416 million hectares in 1978 to 4.5245 million hectares in 2010, while terminal energy consumption increased from 18.13 million tons in 1980 to 263.4485 million in 2010. Guangdong was identified as one of the pilot provinces for national low-carbon economic development in 2010. To this end, the Guangdong government set a goal of reducing carbon dioxide emission per unit of GDP by 35% in 2015 relative to 2005 levels [44]. Land use pattern, a factor that significantly affects energy consumption, is also considered in the government's macro control policy. Therefore, ascertaining the relationship between land use spatial distribution structure and energy-related carbon emission is important for formulating land use policies, which promote efficient energy conservation and emission reduction. 

This study analyzes the relationship between land use spatial distribution structure and energy-related carbon emission intensity in Guangdong from 1996 to 2008. The core components of the research are as follows: (1) The spatial distribution structures of different land use types in Guangdong are analyzed by applying the spatial Lorenz curve and calculating the Gini coefficient. (2) The energy-related carbon emissions in different land use types are calculated based on the IPCC accounting method. (3) The relationship between land use spatial distribution structure and energy-related carbon emission intensity is investigated by regression analysis and cointegration test.

To accurately calculate the energy-related carbon emission in each type of land use, we attempt to establish the corresponding relationships between industrial energy consumption and land use types according to the content of land use status classification in China [45]. Based on the research of Li (2009) and Zhao and Huang (2010), land uses in this study are classified into five different types: agricultural land, industrial land, residential and commercial land, traffic land, and other land (includes lakes, rivers, and special designated land; Table 1) [8, 46].

## 2. Methods and Data Sources

### 2.1. Spatial Lorenz Curve and Gini Coefficient

Numerous methods are used to study the spatial distribution structure of land use. Such approaches include information entropy theory [47–49], geographic information system method combined with remote sensing imagery [50–54], landscape ecology method [55–57], and mathematical methods, such as Markov chain modeling and linear programming [58–62]. However, most of these methods are complicated and provide abstract results [63]. Spatial Lorenz curve analysis combines location entropy with the Lorenz curve concept in economics, making it an intuitive method for studying land use distribution structures [63–65]. The spatial Lorenz curve is quantified by the Gini coefficient, whose value indicates the concentration degree of land use spatial distribution. Thus, on the basis of relevant research, we use the spatial Lorenz curve and Gini coefficient to measure the concentration degree of the spatial distribution structures of different land use types. 

#### 2.1.1. Land Use Spatial Lorenz Curve

**Figure 2:**
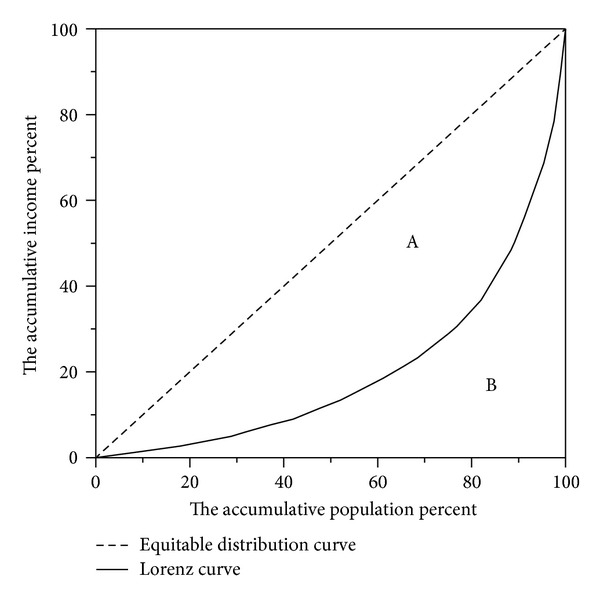
Lorenz curve.

The spatial Lorenz curve of land use can be established by citing two economic concepts, which are Lorenz curve and location quotient. The original Lorenz curve, which comprises population percentage and the corresponding income percentage, is an extensively used tool for examining national wealth distribution [66]. Lorenz curve bends indicate the concentration degree of national wealth and a straight line with no bending translates to equitably distributed wealth (Figure 2) [67]. For intuitive distribution analysis, Lorenz curve is applied to many fields by using relevant percentage variables [68–71]. To measure the spatial distribution of different land use types in this study, the spatial Lorenz curve of land use should comprise specific land percentage and regional area percentage. The specific land percentage refers to the ratio of specific land use area in each city to the whole specific land use area in the province, while the regional area percentage denotes the ratio of each city area to the province area. Actually, the relationship between specific land percentage and regional area percentage can be defined by location quotient in economics [64, 65].

location quotient is the ratio between a special factor percentage and the entire factor percentage. In this study, the special factor percentage is the specific land percentage, and the entire factor percent is the regional area percentage. These percentages are expressed as follows:

(1) $$\begin{array}{c} Q_{ni}=\frac{S_{ni}/\sum S_{ni}}{S_{n}/\sum S_{n}}, \end{array}$$

where *i* and *n* are the indices for land use types and cities in Guangdong, respectively; *Q*_*ni*_ is the location quotient of land use type *i*  (*i* = 1,2, 3,4, 5) in city *n*  (*n* = 1,2, 3,…, 21) (Table 1 and Figure 1); *S*_*ni*_ represents the area occupied by land use type *i* in city *n*; ∑*S*_*ni*_ denotes the area occupied by land use type *i* in Guangdong; *S*_*n*_ represents the area occupied by city *n*; and ∑*S*_*n*_ is the area occupied by Guangdong Province. The numerator represents the area ratio of land use type *i* in city *n*, and the denominator represents the regional area percentage.

**Figure 3:**
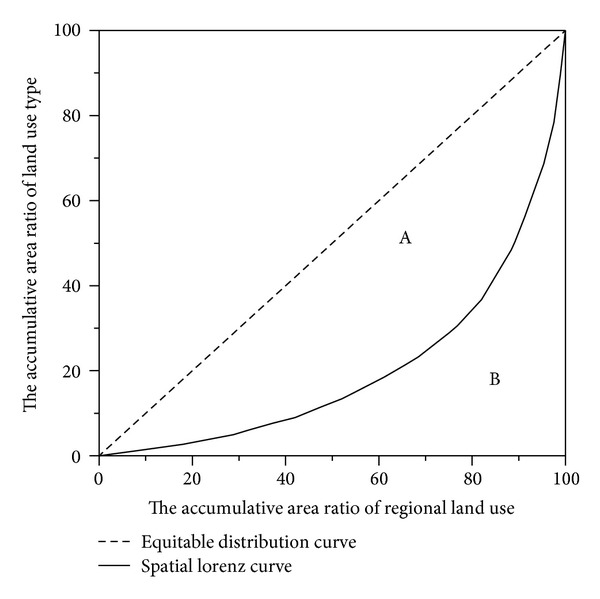
Spatial Lorenz curve.

On the basis of the implications of Lorenz curve, we rank *Q*_*ni*_ from the smallest to the largest; consider an ordinate value as the accumulative area ratio of different land use types and an abscissa value as the accumulative regional area percentage; and then plot the spatial Lorenz curve (Figure 3). The curve bends indicate the concentration degree of land use spatial distribution in the studied region. A straight line with no bending corresponds to equitably distributed land use.

#### 2.1.2. Gini Coefficient

The Gini coefficient measures the land use spatial distribution based on quantifies the bends of spatial Lorenz curve [72]. It is defined as

(2) $$\begin{array}{c} G=\frac{S_{\text{A}}}{S_{\text{A}}+S_{\text{B}}}, \end{array}$$

where *G* is the Gini coefficient, *S*_A_ represents the area between the equitable distribution curve and spatial Lorenz curve (Figure 3, area A), *S*_A_ + *S*_B_ denotes the area under the equitable distribution curve (Figure 3, areas A and B). The Gini coefficient reflects deviation from the equitable distribution curve. In the spatial Lorenz curve, therefore, a high Gini coefficient represents high degree of land use concentration.

We calculate the Gini coefficient in (2) by the curve regression method [73, 74]. When the vertical and horizontal axes are both set to one unit, (2) is converted into

(3) $$\begin{array}{c} G=1-2S_{\text{B}}=1-\sum \left(X_{n}-X_{n-1}\right)\times \left(Y_{n}+Y_{n-1}\right), \end{array}$$

where *X*_*n*_ and *Y*_*n*_ are the indices for the abscissa value and ordinate value of the spatial Lorenz curve, respectively; thus, 0 < *G* ≤ 1. According to the commonly used division standard of the Gini coefficient, land use distribution characteristics can be estimated as follows: when 0 < *G* ≤ 0.2 stands for absolute decentralization, 0.2 < *G* ≤ 0.3 represents decentralization; 0.3 < *G* ≤ 0.4 indicates appropriate concentration; 0.4 < *G* ≤ 0.5 means concentration; and 0.5 < *G* ≤ 1 corresponds to absolute concentration [73].

### 2.2. Energy-Related Carbon Emission Intensity

#### 2.2.1. Calculation of Energy-Related Carbon Emission

Fossil energy accounts for more than 90% of total energy consumption. It is the main source of energy-related carbon emission in China [75]. Energy-related carbon emissions, which include the carbon content of both carbon dioxide and methane, are released from the consumption of fossil and biomass energy. To calculate the fossil energy-related carbon emission both from final consumption and transformational consumption, we separately calculate the carbon emission from final fossil energy consumption and that from terminal thermal power and heating consumption because the majority of fossil energy consumption in the energy conversion process is used to produce thermal power and heating (refer to the energy balance table for Guangdong in the China Energy Statistics Yearbook). The energy-related carbon emissions are calculated as follows [8, 76]:

(4) $$\begin{array}{c} \text{C}{\text{T}}_{i}=\text{C}{\text{R}}_{i}+\text{C}{\text{E}}_{i}+\text{C}{\text{H}}_{i}+\text{C}{\text{B}}_{i}, \end{array}$$

where CT_*i*_ represents the total energy-related carbon emission of land use type *i* (classified in Table 1); CR_*i*_ is the carbon emission from final fossil energy consumption, excluding the thermal power and heating supply for land use type *i*; CE_*i*_ and CH_*i*_ are the indices for the fossil energy-related carbon emissions during the supply of thermal power and heating, respectively, in land use type *i*; CB_*i*_ denotes the carbon emission from noncommercial energy, which is used by the residents of residential and commercial land. 

The detailed calculation methods are as follows:

(5) CRi=∑jQRNij×EFj=∑jQRij×NCVj×EFj×10−3,EFj=Acj×Bcj+Amj×Bmj,

(6) $$\begin{array}{c} \text{C}{\text{E}}_{i}=\text{Q}{\text{E}}_{i}\times \text{EE}\times \text{C}{\text{F}}_{c}, \end{array}$$

(7) $$\begin{array}{c} \text{C}{\text{H}}_{i}=\text{Q}{\text{H}}_{i}\times \text{EH}\times \text{C}{\text{F}}_{c}. \end{array}$$

In (5), CR_*i*_ (10^4^ t) is the carbon emission from final fossil energy consumption, excluding thermal power and heating for land use type *i*; QRN_*ij*_ (TJ) represents the combusted quantity of energy type *j* (*j* refers to the fossil energy types in Table 2) in land use type *i*; QR_*ij*_ (10^4^ t (10^7^ m^3^)) is the consumed fossil energy type *j* in land use type *i*; NCV_*j*_ (TJ/Gg) denotes the net calorific value of fossil energy *j*; QRN_*ij*_ is the product of QR_*ij*_ and NCV_*j*_; EF_*j*_ (t/TJ) is the default carbon emission factor of fossil energy *j*; *A*_*cj*_ and *A*_*mj*_ (t/TJ) represent the default carbon contents in carbon dioxide and methane, respectively; *B*_*cj*_ and *B*_*mj*_ denotes the indices for default oxidation carbon factors; and 10^−3^ is the coefficient of unit conversion. 

The carbon emission from consumed biomass energy (CB_*i*_), which includes biogas, crop stalk, and firewood, can also be calculated by (5). The carbon emission factors are taken from the Provincial Greenhouse Gas List Preparation Guide (Table 2) [77].

In (6) and (7), CE_*i*_ (10^4^ t) and CH_*i*_ (10^4^ t) are the indices for the carbon emissions from thermal power consumption and heating supply, respectively, in land use type *i*. The carbon emissions from thermal power and heating can be calculated by using standard coal consumption given that coal is the main source of thermal power and heating supply in China. QE_*i*_ (10^10^ kWh) and QH_*i*_ (10^13^ kJ) represent the quantities of thermal power consumption and heating supply, respectively, in land use type *i*; EE (g/kWh) and EH (kg/GJ) are the standard coal consumption levels per unit of thermal power and heating supply, respectively; and CF_*c*_ is the carbon emission coefficient of unit standard coal consumption. EE and EH are based on the China Power Statistical Yearbook (1997–2009) (Table 3) [78]. CF_*c*_ equals 0.67 kg C/kgce [79].

#### 2.2.2. Energy-Related Carbon Emission Intensity in Different Land Use Types

The energy-related carbon emission intensity of land use pertains to the energy-related carbon emission in the unit area of land use [8]. It is calculated as follows:

(8) $$\begin{array}{c} \text{C}{\text{I}}_{i}=\frac{\text{C}{\text{T}}_{i}}{{\text{S}}_{i}}, \end{array}$$

where CI_*i*_ (t/hm^2^) represents the energy-related carbon emission intensity in land use type *i*, CT_*i*_ (10^4^ t) denotes the energy-related carbon emission in land use type *i*; and *S*_*i*_ (10^4^ hm^2^) is the area occupied by land use type *i*.

### 2.3. Regression Analysis and Cointegration Test

Regression analysis is commonly used in research on relationships among time series variables. Because of the relevance of time series data, the unit root always exists in time series. Time series with unit roots are called nonstationary time series, and may considerably influence the results of a regression model [80]. Although some time series are nonstationary, certain linear combinations among such series are stationary. This linear combination reflects a stable long-term relationship among variables, and suggest that cointegration among the variables exist. If the unit roots of time series variables are uniformly integrated, the cointegration relationship can be verified by the Engle-Granger testing method [81, 82]. Engle and Granger argue that the residual series of regression models should be stationary if cointegration among variables exists [83]. Thus, the cointegration relationship can be evaluated by determining the stationarity of residual series.

Eliminating inaccurate regression in relationship research entails the following steps for regression analysis and cointegration testing: first, the stationarity of variables is determined by using Augmented Dickey-Fuller (ADF) values. The variables in a uniform integration order are then chosen to build a regression model. Finally, in accordance with the Engle-Granger method, ADF values are used to test the stationarity of the residual series in the regression model. If the residual series exhibit integration of order 1, then cointegration exists in the regression variables and the regression model is reasonable [83].

Studies on the energy-related carbon emission factors in China show that energy-related carbon emission is influenced by multiple factors, including industrial structure, energy consumption structure, energy efficiency, and production efficiency [84–87]. Different land use types are classified by industries and are investigated as separate research objects. These attributes indicate that the influence of structures, including industrial structure and energy consumption structure, are insignificant. Thus, GDP per unit energy consumption (denoted as GE) and GDP per unit capital investment (denoted as GC), as well as land use distribution structure (denoted as LS), are chosen as the independent variables in this study. GE and GC represent energy efficiency and production efficiency, respectively. Considering the effect of area changes on energy-related carbon emission, we choose energy-related carbon emission intensity as the dependent variable, denoted as CI. To eliminate the heteroscedasticity that may exist in time series, we transform the four variables into natural logarithmic form (the logarithm, which is suitable for measuring the changes in elasticity among variables, can eliminate heteroscedasticity without affecting the relationship among variables) and denote these as lnGE, lnGC, lnLS, and lnCI. The relationships among these variables in land use types, except for other land, are determined by multiple regression analysis. The energy-related carbon emission in other land originates from other economic development, which does not relate to certain corresponding industry and value accounting. For this category, therefore, a simple linear regression model is constructed to determine the relationship between land use distribution structure and carbon emission intensity. 

### 2.4. Data sources

Land use data are taken from the Classification Survey Statistics of Land Use for Guangdong (1997–2009), collected by the Guangdong Department of Land and Resources [88]. Energy data are obtained from China Energy Statistical Yearbook (1997–2009) [89] and China New Energy and Renewable Energy Yearbook (2009) [90]. Other data come from Guangdong Statistical Yearbook (1997–2011) [91] and Guangdong Agricultural Statistical Yearbook (1997–2011) [92].

## 3. Results and Discussion

### 3.1. Spatial Distribution Structures of Different Land Use Types

**Figure 4:**
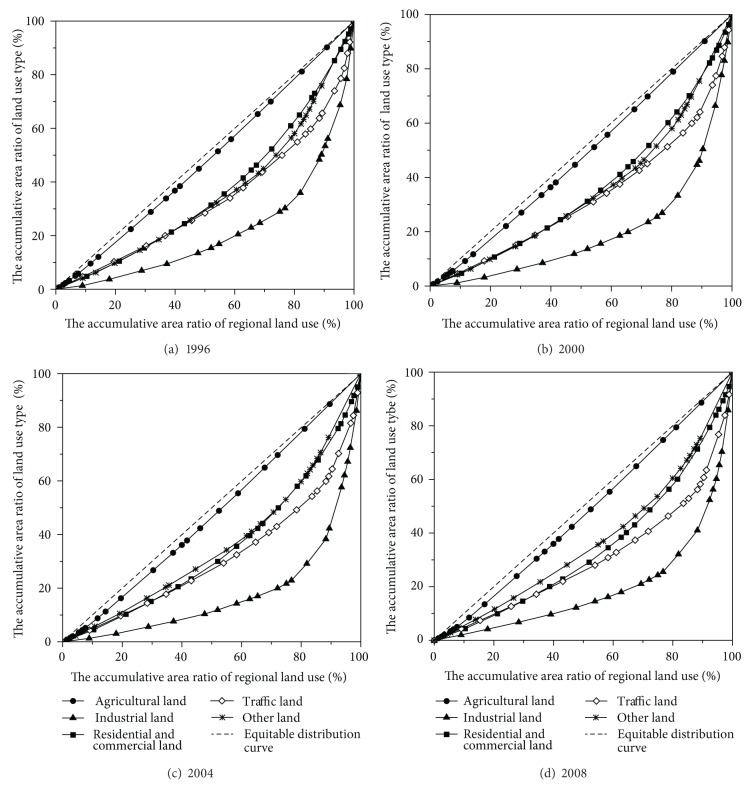
Spatial Lorenz curves of different land use types.

The spatial Lorenz curves of different land use types are shown in Figure 4. The spatial Lorenz curves of agricultural land are always the closest ones to the equitable distribution curve, whereas the industrial land curves are always the farthest. These characteristics indicate that agricultural land exhibits the most decentralized distribution among the five land use types, whereas industrial land shows the most concentrated distribution. Traffic land is more concentrated than residential and commercial land, as well as other land, over 1996 to 2008.

**Figure 5:**
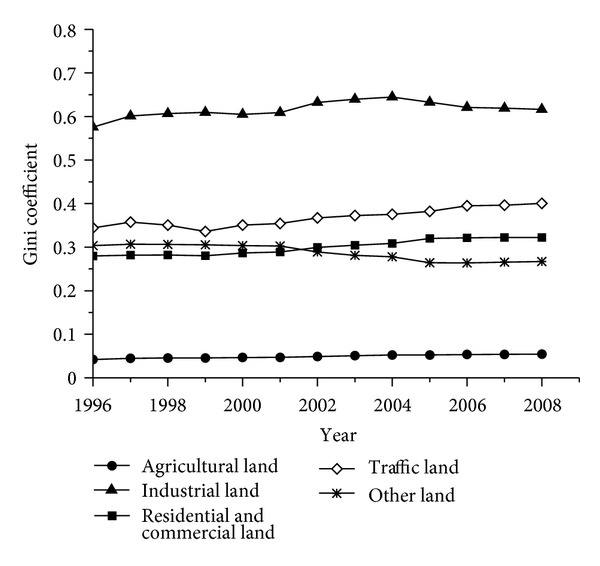
Changes in the Gini coefficients of different land use types from 1996 to 2008.

The Gini coefficients are calculated based on the spatial Lorenz curve to quantify the distribution characteristics of land use. The results are shown in Figure 5 and Table 4. The average Gini coefficient of agricultural land was 0.0491, the smallest among the Gini coefficients of the five land use types. Although the Gini coefficient of agricultural land did not visibly increase in terms of absolute value, its average annual growth rate was higher than that observed for the other land use types. These results indicate that during the study period, agricultural land was absolutely decentralized, with a trend of concentrated use. Industrial land had the highest average Gini coefficient at 0.6163, indicating absolute concentration. Although minimal fluctuation occurs, the increasing trend of industrial land concentration was clearly observable, especially from 2002 to 2004. The average Gini coefficients of residential and commercial land and other land were 0.2999 and 0.2874, respectively, indicating that these two types of land use were distributed in decentralized manners, with different change trends. Residential and commercial land tended toward appropriate concentration, whereas other land tended toward decentralization. Traffic land exhibited appropriate concentration, with an average Gini coefficient of 0.3679; however, this value increased, indicating a concentrated trend for traffic land. 

### 3.2. Energy-Related Carbon Emission Intensity in Different Land Use Types

**Figure 6:**
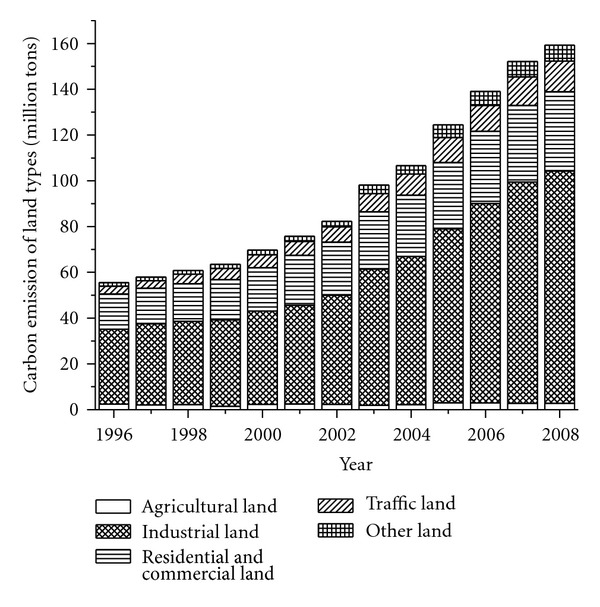
Energy-related carbon emissions in different land use types.

**Figure 7:**
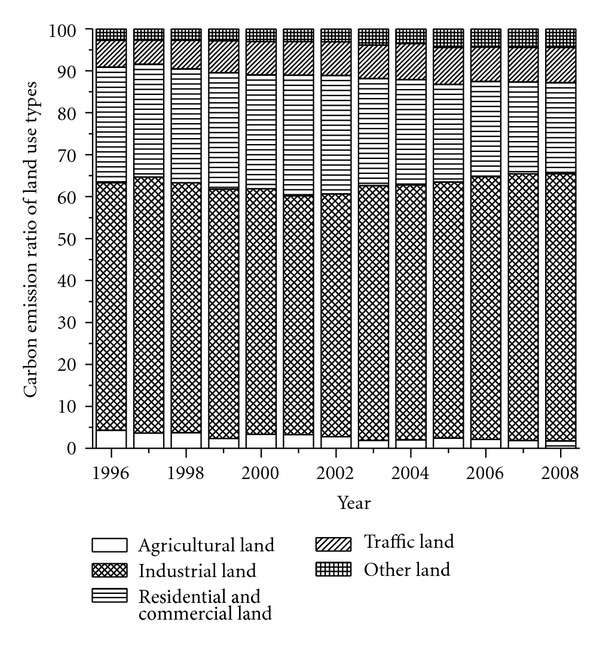
Composition of energy-related carbon emission.

Using the methods discussed in Section 2, we calculate the energy-related carbon emissions in different land use types (Table 5, Figures 6 and 7). During the studied period, the total amount of energy-related carbon emission increased from 55.53 million tons to 159.33 million tons, with the annual increased rate of 9.18%. The energy-related carbon emissions in all land use types significantly increased in varying degrees. The carbon emission in industrial land increased from 32.79 million tons to 101.43 million tons. The proportion of total carbon emission in this land use type also increased from 59.06% to 63.66%, the fastest increase among all the land use types. On the basis of the increase in carbon emission, we rank the rest of the land use types as follows: residential and commercial land > traffic land > other land > agricultural land (Table 5). Although the carbon emissions in residential and commercial land and agricultural land increased 19.29 million tons and 0.49 million tons, respectively, their proportions of total carbon emission decreased by 5.86% and 2.47%, respectively. The carbon emissions in traffic land and other land increased 9.83 million tons and 5.56 million tons, respectively, and their proportions of total carbon emission increased by 2.02% and 1.71%, respectively. These results show that during the studied period, energy-related carbon emission intensively increased in all the land use types. The anthropogenic activities in the other three land use types more considerably increased than those in agricultural land and residential and commercial land. 

Energy-related carbon emission intensities are calculated according to (8). Results are shown in Table 6. For the period 1996 to 2008, the average energy-related carbon emission intensity in Guangdong increased from 3.2326 t/hm^2^ to 9.2525 t/hm^2^, with an annual increase rate of 9.16%. The carbon emission intensities in industrial land, traffic land, and residential and commercial land were higher than the provincial level. In addition, the carbon emission intensities in agricultural land and other land were lower than the average values, and the carbon emission intensity in agricultural land was the lowest among all the land use types. The carbon emission intensities in various land use types increased with different ratios. On the basis of annual increase rates, we rank the land use types as follows: other land > traffic land > residential and commercial land > industrial land > agricultural land. The results indicate that the anthropogenic activities per unit area in each land use type increased, and that the continued development in other land caused a high annual increase rate of energy-related carbon emission in this land use type. 

### 3.3. Regression Analysis and Cointegration Testing of Different Land Use Types

Implementing the steps in Section 2.3, we test the stationarity of each variable using ADF values.  Table 7 shows that the variables in the models of agricultural land, industrial land, residential and commercial land, and other land are integrated in the same order. This integration indicates that stable long-term relationships exist among the variables in each of the four models. Regression models can therefore be constructed using these variables. In the traffic land model, the integration order of lnGC differs from those of the other three variables, indicating that a stable long-term relationship exists among the other three variables. Thus, the other three variables are considered in constructing the traffic land model. 

Five regression models are constructed, with their coefficients shown in Table 8. All of the regression models exhibit good fit, as indicated by the large *R*-square values. The models also pass the *F*-test at highly significant levels.  Table 9 shows that the residual series of the regression models pass the stationarity test. These results indicate that cointegration exists among the variables in each model, and confirm the rationality of the five regressions models. 

As indicated by the five models, land use distribution structure influences energy-related carbon emission intensities at different directions and extents. Aside from industrial land, the spatial distribution Gini coefficients of the other four land use types show a significant positive correlation with carbon emission intensities. These results indicate that concentrated land use distribution increases the carbon emission intensities in agricultural land, residential and commercial land, traffic land, and other land. Although the land structure variable is significant only at 20% level in the statistical *t*-test (*P* value, 0.1722), the model of industrial land also indicates that the Gini coefficient of spatial distribution is negatively correlated with carbon emission intensity in the long term. With respect to extent of influence, every 1% change in the spatial distribution Gini coefficients causes changes of 1.4794%, –1.4262%, 2.4352%, 3.8939%, and 9.7493% in the agricultural land, industrial land, residential and commercial land, traffic land, and other land, respectively. Among the land use types, other land is the type most strongly affected by spatial distribution structure. This result indicates that the other four types of land use, where intensive anthropogenic activities occur, are affected by other factors, such as energy efficiency and production efficiency. Meanwhile, the regression model of other land shows that the spatial distribution structure of land use strongly influences carbon emission intensity when no obvious industrial development and social activities occur in this land use type. 

On the basis of the integrative effects of all the independent variables in the regression models (except for the other land model), we can analyze measures for reducing carbon emission intensity as follows. In the regression model of agricultural land, energy efficiency and product efficiency are negatively correlated with energy-related carbon emission intensity, whereas the concentration degree of spatial distribution is positively correlated with the dependent variable. Thus, improving the efficiency of energy use and production, as well as decentralize the agricultural land distribution can reduce the carbon emission intensity in agricultural land. The industrial land model shows that improving energy efficiency increases the carbon emission intensity in industrial land. This phenomenon also occurs in the residential and commercial land model, in which improving the efficiency of energy utilization and production increases carbon emission intensity (Table 8). Carbon emission per unit land use may increase when production per unit land use rises as a result of improved energy efficiency and production efficiency. Improving energy efficiency and production is the development trend driven by technological progress. Thus, controlling energy-related carbon emission through land use distribution structures is crucial. The coefficients of the models indicate that centralize the industrial land and decentralize the residential and commercial land can reduce the carbon emission intensity, respectively. In traffic land, energy efficiency improvement and land use decentralization both can reduce energy-related carbon emission intensity. 

The analyses above indicate that adjusting the spatial distribution structure of land use is important in reducing the energy-related carbon emission intensities in all land use types. The coefficients shown in Table 8 indicate that among all the variables in each model, the spatial distribution structure of land use has the largest effect on carbon emission intensity. Thus, focusing on efficient spatial distribution structure of land use can effectively controls carbon emission. 

## 4. Conclusion 

The spatial distribution structure of land use influences energy consumption patterns and is one of the most important factors for regional energy-related carbon emission. However, studies on the relationship between land use spatial distribution structure and energy-related carbon emission are rare. The current work discusses this relationship for different land use types in Guangdong, an economically advanced region in China and has been chosen as a pilot province for national low-carbon development. On the basis of land use pattern and industry types, land use is classified into five types which contain agricultural land, industrial land, residential and commercial land, traffic land, and other land. The spatial distribution structures of different land use types are analyzed by using the spatial Lorenz curve and Gini coefficient. The corresponding energy-related carbon emissions are calculated with factors taken from the Provincial Greenhouse Gas List Preparation Guide [77]. In constructing regression models, the energy-related carbon emission intensities in different land use types are set as dependent variables. Energy efficiency, production efficiency, and land use spatial distribution structure are chosen as independent variables. According to the results form regression analysis and cointegration testing, we draw the following conclusions. (1) Aside from other land, the other four types of land use displayed different concentration trends during 1996 to 2008. Among the five types of land use, industrial land was the most concentrated, whereas agricultural land was the most decentralized but exhibits the fastest change rate. (2) During the study period, energy-related carbon emission in each type of land use considerably increased. The anthropogenic activities in industrial land still account for the highest energy consumption. (3) Spatial distribution concentration is negatively correlated with energy-related carbon emission intensity in industrial land in the long term; by contrast, these two variables show a positive correlation in agricultural land, residential and commercial land, traffic land, and other land. These results indicate that the centralized distribution of industrial land may reduce the corresponding carbon emission intensity, but that the centralized distribution of the other four land use types can increase carbon emission intensity. (4) Land use spatial distribution structure has a more intensive effect on carbon emission intensity than energy efficiency and production efficiency do. 

Thus, adjusting the spatial distribution structure of land use is an effective way to control carbon emission. The findings provide valuable reference to develop comprehensive policies for regional energy conservation and carbon emission reduction at a new perspective. Meanwhile, some of the related suggestion can be summarized as follows. (1) Strengthening the intensive use of agricultural land by improving the input-output efficiency of energy, labor, and capital. Ensure the spatial distribution of agricultural land keep on decentralizing when the land is occupied and compensated by other land types or adjusted in internal structure. (2) Promoting industrial agglomeration and unify the layout of energy-intensive industries coordinately. Meanwhile, technological innovation application, infrastructure construction improvement, and recycling economy should be encouraged in industrial development to make sure that resource utilization and scale economy effects are sufficiently developed. (3) Guiding the residents and commercial industry expand or transfer from population intensive region to the relative sparse region, avoiding the unnecessary energy waste from intensive energy consumption and the low commute efficiency caused by excessive concentration of residential and commercial land. (4) Increasing the density of road network in underdeveloped regions of Guangdong. Adjusting the transport instrument structure and industrial management standards, improve the traffic efficiency and reduce the unnecessary repeatedly energy cost by passengers and freight transport in the region of sparse road network. (5) Improving the development efficiency in other land by formulating specification management system. Trying to develop the other land evenly in spatial distribution instead of concentrated develop.

Although the results in this study are credible and representative, the study has certain limitations. Aside from the different spatial distributions of various land use types, the land use factors that affect regional energy consumption patterns also include land use composition. Thus, the result of this empirical study does not represent the common mechanism in other regions. In the future study, coordinating the internal relationship among land use factors and discussing their integrated effects on regional energy-related carbon emission are more comprehensive ways to define the mechanism of how the changes in land use influence regional energy-related carbon emission. Another limitation is the difficult access to land use classification data. Collecting land use area data on earlier years by land use classification survey is difficult, and accurately identifying different land use types through Landsat TM images with a 30 m resolution is very hard [27]. We anticipate the collection of more relevant land use data on earlier years, so that the argument in this study can be validated for longer time series. 

## Figures and Tables

**Table 1:** Classification of land use types and their corresponding relationship with industrial energy consumption.

Land use type	Specific types	Corresponding industrial energy consumption
Agricultural land	Cultivated land, orchard land, forest land, grassland, and other agricultural lands	Farming, forestry, animal husbandry, fishery
Industrial land	Independent industrial and mining lands	Industry
Residential and commercial land	Urban area, rural-residential areas	Construction, wholesale and retail trade, catering services, residential living
Traffic land	Traffic land	Transportation, storage, postal and telecommunications services
Other land	Unused land and other land (e.g., special designated land, lakes, and rivers)	Other industries

**Table 2:** Net calorific values and default carbon emission factors of the considered energy types.

Energy type	NCV_*j*_ (TJ/Gg)	EF_*j*_ (t/TJ)
Raw coal	20.91	25.64
Cleaned coal	26.34	24.89
Other washed coal	8.36	24.89
Briquettes	20.91	30.24
Coke	28.44	27.44
Coke oven gas (MJ/m^3^)	16.73	12.93
Other gas types (MJ/m^3^)	22.14	12.93
Crude oil	41.82	19.70
Gasoline	43.07	18.52
Kerosene	43.07	18.52
Diesel oil	42.65	19.80
Fuel oil	41.82	20.68
Liquefied petroleum gas	50.18	16.86
Refinery gas	46.06	17.84
Natural gas (MJ/m^3^)	38.93	15.15
Other petroleum products	41.82	19.60
Other coking products	28.44	27.44
Other energy types	29.31	12.20
Biogas (MJ/m^3^)	20.91	14.89
Crop stalk	12.55	30.57
Firewood	16.73	30.57

**Table 3:** Standard coal consumption per unit of thermal power and heating supply.

Year	Standard coal consumption per unit of thermal power (g/kWh)	Standard coal consumption per unit of heating supply (kg/GJ)
1996	392	40.13
1997	386	40.77
1998	381	40.39
1999	372	41.22
2000	372	39.70
2001	363	40.08
2002	358	40.48
2003	358	40.64
2004	357	40.22
2005	353	40.24
2006	352	40.32
2007	343	40.50
2008	336	40.14

Data are obtained from China Power Statistical Yearbooks.

**Table 4:** Spatial distribution structures and divisions of different land use types.

Year	Agricultural land	Industrial land	Residential and commercial land	Traffic land	Other land
1996	0.0422	0.5757	0.2799	0.3441	0.3037
1997	0.0447	0.6011	0.2817	0.3575	0.3066
1998	0.0456	0.6068	0.2819	0.3508	0.3065
1999	0.0458	0.6094	0.2802	0.3359	0.3056
2000	0.0464	0.6047	0.2867	0.3506	0.3035
2001	0.0469	0.6089	0.2891	0.3545	0.3027
2002	0.0491	0.6322	0.2995	0.3671	0.2890
2003	0.0509	0.6395	0.3047	0.3727	0.2810
2004	0.0525	0.6447	0.3087	0.3752	0.2780
2005	0.0524	0.6327	0.3201	0.3820	0.2642
2006	0.0533	0.6208	0.3213	0.3949	0.2636
2007	0.0541	0.6191	0.3222	0.3964	0.2655
2008	0.0545	0.6164	0.3223	0.4006	0.2667
Average value	0.0491	0.6163	0.2999	0.3679	0.2874
Annual change rate	2.15%	0.57%	1.18%	1.28%	−1.08%
Distribution division	Absolute decentralization	Absolute concentration	Decentralization	Appropriate concentration	Decentralization

**Table 5:** Energy-related carbon emissions in different land use types during 1996–2008 (million ton).

Year	Agricultural land	Industrial land	Residential and commercial land	Traffic land	Other land	Total
1996	2.36	32.79	15.32	3.54	1.51	55.53
1997	2.11	35.35	15.57	3.36	1.54	57.93
1998	2.25	36.30	16.54	4.08	1.71	60.88
1999	1.52	37.74	17.62	4.82	1.81	63.52
2000	2.35	40.80	18.93	5.55	2.12	69.75
2001	2.45	43.15	21.80	6.06	2.31	75.76
2002	2.29	47.67	23.27	6.58	2.54	82.36
2003	1.88	59.58	25.06	7.83	3.80	98.16
2004	2.13	64.80	26.85	9.10	3.75	106.62
2005	3.02	75.97	29.02	10.94	5.51	124.45
2006	2.91	87.17	31.57	11.27	6.12	139.04
2007	2.83	96.70	33.42	12.44	6.76	152.15
2008	2.85	101.43	34.61	13.37	7.07	159.33
Change value	0.49	68.64	19.29	9.83	5.56	103.80
Annual increased rate	1.58%	9.87%	7.03%	11.71%	13.73%	9.18%

**Table 6:** Carbon intensities in different land use types from 1996 to 2008 (t/hm^2^).

Year	Agricultural land	Industrial land	Residential and commercial land	Traffic land	Other land	Average value
1996	0.1604	147.8420	14.5272	45.1691	1.3999	3.2326
1997	0.1439	142.0132	14.5558	39.2389	1.4306	3.3740
1998	0.1536	138.4803	15.3286	44.6711	1.5923	3.5453
1999	0.1035	139.1317	16.1736	49.7709	1.7002	3.6992
2000	0.1608	150.8519	17.1461	54.5343	1.9975	4.0613
2001	0.1675	156.1487	19.6515	58.7774	2.1658	4.4113
2002	0.1574	156.5664	20.5831	61.7720	2.3601	4.7923
2003	0.1296	179.9739	21.9476	72.3479	3.4971	5.7068
2004	0.1467	184.5160	23.3094	82.5935	3.4396	6.1973
2005	0.2079	220.9347	24.4428	96.8572	5.1895	7.2319
2006	0.2006	237.5971	26.3965	95.8099	5.7947	8.0751
2007	0.1959	254.4186	27.7520	103.5313	6.4395	8.8357
2008	0.1969	262.7841	28.6123	110.0966	6.7675	9.2525
Annual increased rate	1.73%	4.91%	5.81%	7.71%	14.03%	9.16%

**Table 7:** ADF values and integration orders of variables in each model.

Model	Agricultural land		Industrial land		Residential and commercial land		Traffic land		Other land	
variables	ADF value	Integration order	ADF value	Integration order	ADF value	Integration order	ADF value	Integration order	ADF value	Integration order
lnGE	−7.0353***	1	−3.3655**	1	−4.2561***	1	−3.9848**	1	—	—
lnGC	−5.6262***	1	−3.6838**	1	−3.5406**	1	−4.6038***	0	—	—
lnLS	−3.3381**	1	−2.7999*	1	−2.7910**	1	−3.6112**	1	−3.8533*	1
lnCI	−6.3699***	1	−3.6165*	1	−3.7370**	1	−5.9159***	1	−5.1981***	1

Notes: *, **, *** denote significance at the 10%, 5%, and 1% levels, respectively.

**Table 8:** Coefficients of the five models.

Variables	Agricultural land	Industrial land	Residential and commercial land	Traffic land	Other land
Intercept	4.3221***	5.6705***	5.4749***	7.6108***	10.8168***
lnGE	−1.0860***	1.3714***	0.7125**	–1.5946***	—
lnGC	−0.0869***	−0.3723***	0.1493	—	—
lnLS	1.4794***	−1.4262	2.4352***	3.8939***	9.7493***
*R*-square	0.9910	0.9354	0.9767	0.9773	0.8956
*F*-test value	330.3216***	43.45113***	125.6984***	215.4236***	94.4115***

Notes: *, **, *** denote significance at the 10%, 5%, and 1% levels, respectively.

**Table 9:** Stationarity test results for the residual series in the regression models.

Models	Agricultural land	Industrial land	Residential and commercial land	Traffic land	Other land
ADF value	−3.5780	−3.2991	−5.1956	−2.1985	−2.9509
*P* value	0.0019	0.0034	0.0001	0.0322	0.0069
Stationarity	Stationary	Stationary	Stationary	Stationary	Stationary

**Table 10:** Energy consumption in different types of land use (10^4^ ton standard coal equivalent).

Year	Agricultural land	Industrial land	Residential and commercial land	Traffic land	Other land
1996	368.27	4824.86	2240.35	613.05	229.93
1997	334.65	5234.19	2285.91	583.35	233.29
1998	355.70	5407.87	2414.26	699.89	258.99
1999	245.40	5709.13	2586.78	839.21	274.21
2000	370.75	6110.75	2785.05	965.75	320.64
2001	385.33	6482.84	3199.58	1054.31	349.43
2002	360.55	7177.19	3409.13	1146.83	384.88
2003	298.69	8938.86	3698.44	1351.97	573.72
2004	336.92	9938.45	3974.78	1568.56	567.12
2005	470.63	11664.64	4361.33	1922.88	836.03
2006	452.46	13445.77	4674.41	1982.17	927.34
2007	439.06	14845.45	5022.51	2186.92	1023.54
2008	441.42	15510.91	5226.68	2352.14	1069.61

**Table 11:** Input capital in different types of land use (100 million Yuan).

Year	Agricultural land	Industrial land	Residential and commercial land	Traffic land
1996	5.39	457.08	384.15	340.11
1997	4.71	302.15	369.33	211.97
1998	7.65	338.07	469.65	241.12
1999	7.05	393.15	558.52	270.35
2000	8.85	321.29	548.14	283.32
2001	7.47	390.22	533.44	283.36
2002	6.09	542.35	511.88	278.79
2003	9.07	685.92	767.83	351.90
2004	7.33	938.74	887.95	404.88
2005	12.29	1602.37	1132.19	498.70
2006	14.38	1853.86	1118.69	574.98
2007	21.01	1918.81	1275.85	619.81
2008	23.98	1862.23	1423.45	820.22

**Table 12:** GDP in different types of land use (100 million Yuan).

Year	Agricultural land	Industrial land	Residential and commercial land	Traffic land
1996	954.71	2788.82	2277.93	497.68
1997	999.32	3191.03	2520.99	543.15
1998	1037.09	3603.30	2782.94	579.51
1999	1077.65	3996.54	3095.60	612.74
2000	1102.55	4526.20	3504.51	718.48
2001	1126.93	5033.99	3925.78	818.73
2002	1175.49	5783.63	4416.51	870.32
2003	1201.67	7000.60	4916.92	923.46
2004	1251.06	8420.89	5507.29	1035.12
2005	1312.66	9753.01	6260.53	1210.12
2006	1367.72	11472.51	7101.13	1385.19
2007	1411.73	13508.44	8051.59	1518.51
2008	1463.96	15163.55	8782.34	1625.17

## Appendix

See Tables 10, 11, and 12.
